# Understanding the Relationship between Activity and Neighbourhoods (URBAN) Study: research design and methodology

**DOI:** 10.1186/1471-2458-9-224

**Published:** 2009-07-10

**Authors:** Hannah M Badland, Grant M Schofield, Karen Witten, Philip J Schluter, Suzanne Mavoa, Robin A Kearns, Erica A Hinckson, Melody Oliver, Hector Kaiwai, Victoria G Jensen, Christina Ergler, Leslie McGrath, Julia McPhee

**Affiliations:** 1 Centre for Physical Activity and Nutrition Research, Auckland University of Technology, Auckland, New Zealand; 2 Centre for Social and Health Outcomes Research and Evaluation, Massey University, Auckland, New Zealand; 3 School of Public Health and Psychosocial Studies, Auckland University of Technology, Auckland, New Zealand and School of Nursing and Midwifery, The University of Queensland, Qld 4072, Australia; 4 School of Geography, Geology, and Environmental Science, University of Auckland, Auckland, New Zealand; 5 Whariki Research Group, Massey University, Auckland, New Zealand

## Abstract

**Background:**

Built environment attributes are recognized as being important contributors to physical activity (PA) engagement and body size in adults and children. However, much of the existing research in this emergent public health field is hindered by methodological limitations, including: population and site homogeneity, reliance on self-report measures, aggregated measures of PA, and inadequate statistical modeling. As an integral component of multi-country collaborative research, the Understanding the Relationship between Activity and Neighbourhoods (URBAN) Study seeks to overcome these limitations by determining the strengths of association between detailed measures of the neighborhood built environment with PA levels across multiple domains and body size measures in adults and children. This article outlines the research protocol developed for the URBAN Study.

**Methods and design:**

The URBAN Study is a multi-centered, stratified, cross-sectional research design, collecting data across four New Zealand cities. Within each city, 12 neighborhoods were identified and selected for investigation based on higher or lower walkability and Māori demographic attributes. Neighborhoods were selected to ensure equal representation of these characteristics. Within each selected neighborhood, 42 households are being randomly selected and an adult and child (where possible) recruited into the study. Data collection includes: objective and self-reported PA engagement, neighborhood perceptions, demographics, and body size measures. The study was designed to recruit approximately 2,000 adults and 250 children into the project. Other aspects of the study include photovoice, which is a qualitative assessment of built environment features associated with PA engagement, an audit of the neighborhood streetscape environment, and an individualized neighborhood walkability profile centered on each participant's residential address. Multilevel modeling will be used to examine the individual-level and neighborhood-level relationships with PA engagement and body size.

**Discussion:**

The URBAN Study is applying a novel scientifically robust research design to provide urgently needed epidemiological information regarding the associations between the built environment and health outcomes. The findings will contribute to a larger, international initiative in which similar neighborhood selection and PA measurement procedures are utilized across eight countries. Accordingly, this study directly addresses the international priority issues of increasing PA engagement and decreasing obesity levels.

## Background

Increasing physical activity (PA) engagement and reducing obesity levels at the population-level have been identified as national [1] and international [2,3] health priorities. Multiple factors at different levels, including personal, family, social, environmental, and economic attributes, have been shown to influence PA and obesity patterns [4]. Environmental determinants, such as changes in urbanization patterns and the built environment, increased used of labor-saving devices, greater participation in sedentary activities, and reliance on automobiles for transport are now being recognized as key contributors to these health outcomes [2,3,5]. Urban sprawl, a composite measure of many built environment elements, has also been positively related to population-level overweight/obese status [6-8], potentially through reduced accumulation of PA via increased reliance on cars and reduced access to local destinations and public transport infrastructure. Despite these emerging relationships, studies in this field often have methodological flaws that limit the robustness of the findings. More rigorous data are urgently required to inform decision-makers of the built environment variables with greatest potential for improving PA and obesity outcomes.

A number of built environment features have been consistently identified as promoting PA in both adults [9-11] and children [12,13]. For adults these include increased street network connectivity, higher residential population density, greater access to public spaces, shops, and services, and higher levels of mixed land use [14-17]. Adult PA levels are also influenced by streetscape characteristics including neighborhood aesthetics, green space, pedestrian infrastructure, and safety factors [16-20]. For children, distance to school [21], neighborhood design [13,22], traffic safety [23], and access to green spaces [24] and recreation locations [12] have been associated with PA engagement.

Although this evidence is accumulating, there are limitations in many of the studies on which it is based. To date, the majority of research investigating built environment variables with PA engagement and body size has relied on self-report measures, largely drawn from adult samples based in the United States and Australia. Although self-report measures are practical to implement, they do not accurately detect incidental PA accumulation [25] and are affected by recall bias in adults [25] and children [26]. Also, neighborhoods have often not been selected to maximize variation in built environment attributes. Capturing neighborhood variability is fundamental to understanding the magnitude of built environment effects on individual PA engagement within these communities.

Ethnic differences in relation to built environment variables have also been understudied. Within New Zealand, Māori (New Zealand's indigenous people) have higher obesity rates when compared with New Zealand Pākehā/European [27], and it is unknown whether urban form characteristics may influence these groups differently. Furthermore, little research has been conducted with children in this context; yet given the more sporadic nature of PA engagement displayed by children when compared with adults, it is conceivable that built environment variables associated with PA and body size will differ for adults and children. Parental perceptions of neighborhood safety (e.g., stranger danger, traffic concerns) may also be an important influencing factor regarding children's PA engagement [23]. Ignoring these important variables is likely to have resulted in population and site homogeneity, which in turn, may lead to underestimation of effect sizes in associations between the built environment and health outcomes. A further limitation of existing research in this field has been the use of rudimentary analytical techniques that ignore clustering and the multilevel or hierarchical structure of data on individuals living in different households, neighborhoods, cities, and countries. Multilevel modeling that can simultaneously account for factors at individual and neighborhood levels is likely to provide a more robust and sophisticated understanding of PA and health determinants [28].

The International Physical Activity and Environment Network (IPEN) study was set up to overcome the limitations inherent with many previous studies and to address the paucity of rigorous scientific evidence available in this field (refer ). Key strengths of the collaborative study are the: multi-country participation (Australia, Belgium, Colombia, Czech Republic, Hong Kong, New Zealand, United Kingdom, United States of America) to ensure inclusion of diverse urban environments, and the use of standardized protocols to measure the built environment (geographical information systems (GIS)), PA engagement (International Physical Activity Questionnaire – Long Form (IPAQ-LF), accelerometry), and other health outcomes (body size). Once collected, participant and neighborhood data will be combined to facilitate intra-and inter-country multilevel comparisons of built environment, PA, and health outcomes. This will produce more accurate effect size estimations, and improve understanding of international associations between the urban design, PA, and body size status. Purposefully stratifying neighborhoods based on built environment attributes and combining data from multiple sites in diverse countries will ensure that a larger variation of environmental attributes will be gained than those available from any one country.

The Understanding the Relationship Between Activity and Neighbourhood (URBAN) Study contributes to this collaboration by collecting New Zealand-specific built environment and health data from four diverse cities in accordance with IPEN protocols. In addition to the design strengths of the IPEN collaboration, the URBAN Study has incorporated several additional features that will add to its potential to contribute to understanding in this field: a child sample, stratifying neighborhoods by walkability and ethnicity, door-to-door recruitment of participants, streetscape audits, in depth assessments of the perceived environment (via photovoice), and individualized walkability profiles based on participants' residential location. This paper outlines the methods developed for use in the URBAN Study.

## Methods

### Study aim

The overarching aim of the URBAN Study is to understand the associations between neighborhood built environment variables, PA engagement, and body size. Measures of neighborhood urban design, PA levels across multiple domains (leisure, transport, habitual, and overall), and body size will be used to model the associations and establish effect sizes in a diverse sample using apposite statistical modeling.

### Study design

**Figure 1:**
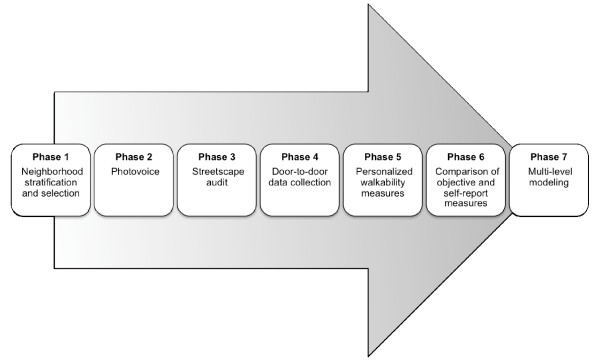
**Diagram of the overall research design for the URBAN Study**.

This research is a cross-sectional study that examines the associations between neighborhood urban design, PA levels, and body size in adults and children residing in selected neighborhoods within four cities in New Zealand (North Shore, Waitakere, Wellington, and Christchurch). The sites were selected for their geographical diversity and because of existing access to city-level GIS data. The study was conceptualized using a multilevel framework, with the levels being: country, city, neighborhood, household, and individual. The URBAN Study is being conducted in seven phases, where each phase informs the subsequent stages of the research (Figure 1). Recruitment for the URBAN Study commenced in April 2008 in North Shore City, and the project uses a rolling data collection process across the four cities; it is anticipated that it will take one and a half years to complete the door-to-door data collection component of the study (phase 4). The host institutions of the research granted ethical approval for the outlined study procedures (AUTEC: 07/126, MUHECN: 07/045).

#### Phase 1: Neighborhood stratification

Within each of the four cities, 12 neighborhoods were selected for investigation. In order to select neighborhoods, a walkability index was created and the domiciled Māori population was estimated. These values were applied to each mesh-block within the cities' boundaries. A mesh-block is a geographic census unit of approximately 100 households constructed by Statistics New Zealand [29]. The walkability index was calculated using combined measures of street connectivity, dwelling density, land use mix, and retail floor area ratio, and was generated using GIS software, ArcInfo 9.1 (ESRI, Redlands, CA). The construction of these measures replicates existing IPEN research procedures [10,30]. Each of the walkability variables is discussed below.

#### • Street connectivity

Street connectivity was estimated by calculating intersection density. Street intersections with three or more unique intersecting streets were extracted from road network data. Mesh-block boundaries are typically defined by street centerlines. Therefore, to ensure that street intersections coincidental with mesh-block boundaries were included, intersection density was calculated as the number of intersections per square kilometer within 20 meters of each mesh-block boundary. Values for each mesh-block were between 0 and 1, where a score closer to 1 indicated higher street connectivity.

#### • Dwelling density

The number of dwellings was estimated using mesh-block data for the number of occupied private dwellings taken from the New Zealand 2006 census [29]. Residential land area was obtained from the land use and zoning data provided by the territorial authorities. Dwelling density was calculated by dividing the number of dwellings by the residential land area for each mesh-block.

#### • Mixed land use

The land use and zoning data were used to categorize land uses into commercial, residential, industrial, open space, and other within each mesh-block. The land use mix was calculated using an entropy index [31], where 0 indicates homogeneity of land use, and a value closer to 1 specified greater heterogeneity of land uses.

#### • Retail floor area ratio

The retail floor area was determined by using building outline data sourced from the territorial authorities. The net retail area was then calculated by dividing the retail floor area by the total retail parcel area within each mesh-block [10]. A higher value indicated less parcel space allocated to car parking at retail sites within the mesh-block.

#### • Walkability index

The walkability index was calculated separately for each city using the above four measures (street connectivity, dwelling density, mixed land use, and retail floor area ratio). The measures were classified into deciles and recoded into values from 1 (1^st ^decile) to 10 (10^th ^decile). The walkability index for each mesh-block was calculated by summing the four 1 to 10 scores, resulting in a possible score from 4 to 40.

#### • Māori population

Distribution of usual Māori residents domiciled within each mesh-block within the four cities was estimated by using 2006 census data [29]. Following the walkability index procedures, the mesh-block Māori population density was classified into deciles and recoded into values from 1 (1^st ^decile) to 10 (10^th ^decile) for each city. Māori comprise 14.6% of the resident population. They are the second-largest ethnic group (after New Zealand Pākehā/European) in New Zealand [32].

#### • Neighborhood selection

Within each city, walkability and Māori population density were each partitioned into tertiles (lower (deciles 1–3), middle (deciles 4–7), and higher (deciles 8–10)). In the interest of capturing variability, only mesh-blocks with higher walkability and higher Māori population density, higher walkability and lower Māori population density, lower walkability and higher Māori population density, and lower walkability and lower Māori population density were eligible for this study. The middle tertile was removed from further analysis at this point.

All eligible mesh-blocks were then identified on city maps and clusters of five contiguous mesh-blocks of similar walkability and/or Māori population density characteristics were grouped together to form neighborhoods. The research team then purposefully selected three neighborhoods for each walkability/Māori population strata per city. This ensured geographical spread within each region and diversity across cities were captured. In total, 12 neighborhoods were selected per city and 48 neighborhoods were chosen across New Zealand. All neighborhoods are drawn from urban settings. In the instances where the number of potential respondents is exhausted within the neighborhood during the door-to-door recruitment phase (generally because of a high number of commercial premises within that setting), an additional contiguous mesh-block of similar built environment and Māori population characteristics is added to the neighborhood.

#### Phase 2: Photovoice

Photovoice is a research method that allows individuals, including those who may be marginalized, to conceptualize their environment through photography. In this study, neighborhood features associated with PA engagement across different cities, settings, and populations are qualitatively captured by photovoice. Children, as well as adults (approximately n = 10 per group) are drawn from five diverse neighborhoods in North Shore and Waitakere cities and invited to participate in the photovoice component of the URBAN Study. These participants do not necessarily partake in the door-to-door data collection aspect of the study. After an initial briefing, participants are each provided with a disposable camera to take photographs of features in their local environment they perceive make their self-defined neighborhood more and less conducive for engaging in PA. The photographs are developed, brought to a participant focus group (either adult- or child-specific). Participants presented noteworthy photos in relation to neighborhood PA attributes and explained the images to the group, both verbally and by way of captions written underneath the pictures. This process, either in small breakout groups or as a whole group discussion, enables the identification of key PA themes of significance and concern for participants in each locality. The discussions are audio taped, transcribed, and thematic induction analyses is conducted using Nvivo software (QSR, VIC, Australia). The photovoice procedures follow an established methodology [33,34].

#### Phase 3: Streetscape audit

In 12 selected street segments in each study neighborhood a streetscape audit using a modified version of the Systematic Pedestrian and Cycling Environment Scan (SPACES) tool [35] is undertaken to assess the presence and absence of features that support walking and cycling (e.g., physical infrastructure, aesthetics, traffic safety attributes). The SPACES, developed in Australia, has demonstrated appropriate reliability for most variables examined in that setting (kappa ≥ 75% agreement) [36], and was adapted for the New Zealand context. The starting point for the audit is randomly selected within the neighborhood and thereafter the street segments are selected sequentially. Scores from each street segment are combined to provide a neighborhood streetscape value. All streetscape audits are conducted when door-to-door data collection is occurring in the city. For reliability purposes, 10% of the street segments are re-audited by a second trained assessor. A training manual based on the SPACES protocols was developed for the URBAN Study that included New Zealand specific reference images.

#### Phase 4: Participant recruitment and data collection

Trained interviewers recruited participants using a door-to-door recruitment strategy. For each selected neighborhood, GIS is used to generate street maps, identify parcel lots, random start points, walk paths, and enumerate households. These maps are provided to three or four trained interviewers for door-to-door recruitment with instructions to approach every n^th ^household. The household sampling rate is determined by dividing the neighborhood dwelling density [29] by the estimated response rate of 60%. This value varies between neighborhoods because of the changeable number of residential dwellings located within each mesh-block. Commercial or temporary residential (e.g., motel rooms) premises are excluded from the sampling frame.

Interviewers start from GIS-derived randomly selected start points and approach the households identified by the enumeration process. The interviewers follow the pre-determined walk path for each neighborhood. Forty-two households are selected in each neighborhood, and one adult and one child (where possible) are surveyed per household. This sampling strategy is designed to yield a total of 2,000 adult participants once data collection is complete. It is estimated that 250 children will be recruited into the study.

Individuals aged between 20–65 years and 3–12 years inclusive usually resident in private dwellings in the 48 selected neighborhoods are eligible to participate in the study. Where there is more than one eligible person in the household, potential participants are identified by the criterion of having the next birthday. Exclusion criteria are: falling outside the age ranges, not intending on living in the household over the measurement period, not resident in the dwelling three months prior to recruitment, unable to speak the English language, or having walking mobility restrictions, such as using crutches. The eligible child in the household is unable to participate in the research if the eligible adult from the household refuses to take part in the study. In the event that there is no eligible adult residing in the household or the eligible adult refuses to participate, the household becomes 'closed' and the interviewer moves on to the next household identified on the neighborhood walk. If no one is at home or an eligible adult resides in the household, but is not available, the interviewer makes a maximum of five return visits for recruitment purposes. The outcome for each visit is recorded on a door-to-door call sheet. Information regarding door-to-door recruitment procedures is documented in a training manual and briefing session.

**Figure 2:**
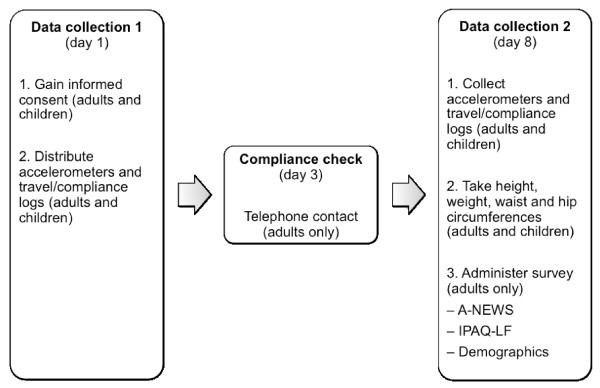
**Measurement battery for the door-to-door component of the URBAN Study**.

Once participants are recruited, two data collection points (data collection 1, data collection 2) are arranged eight days apart, providing a seven-day measurement period (Figure 2). At data collection 1 the interviewer introduces the study, gains informed consent/assent, and distributes the accelerometer and travel/compliance log. Data collection 1 is frequently undertaken at the point of participant recruitment. The interviewer telephones the adult participants three days after data collection 1 to monitor accelerometer compliance. At data collection 2, the interviewer collects the accelerometer and travel/compliance log, measures participants' height, weight, waist and hip circumferences, and conducts the survey with the adult participant. The interviewer follows the same call back recruitment procedures if the participant is not home for data collection 2.

The interviewer enters all information directly on to a personal digital assistant at both time points and subsequently exports the data into Microsoft Excel (Microsoft Corp, Redmond, WA) at the research centre. Quality control audits are conducted on 10% of all interviews by the fieldwork supervisor.

#### • Objective PA measures

PA is measured objectively in adults and children for seven consecutive days using hip-mounted Actical accelerometers (Mini-Mitter, Sunriver, OR) fitted to a purpose-built elastic waistband. The units have been shown to be reliable and valid for these populations [37-39]. Prior to distribution, the accelerometer supervisor prepares the units, including date stamping the devices and setting up the units to record PA and step counts in 30-second epochs. Accelerometers are distributed at data collection 1 by the interviewer and participants are instructed to wear the units for all waking hours for one week (seven days), but remove the monitors when participating in water-based activities. Accelerometers are collected as close as possible to eight days later at data collection 2 by the interviewer. Accelerometers are returned to the research centre and the data are downloaded into Microsoft Excel by the accelerometer supervisor. Once cleaned, data from the unit are included for further analyses if at least 10 hours of data are gathered per day, for a minimum of 5 days. This is in accordance with IPEN protocols [40]. A manual has been developed regarding accelerometer downloading and uploading procedures, data storage, and data cleaning treatment protocols, and automated data extraction procedures are currently being developed. For most analyses, the outcome variables for adults and children will be the percentage of waking time spent in sedentary, light, moderate, and vigorous PA [41].

#### • Travel/compliance log

Participants self-complete a travel and compliance log for the duration of the accelerometer data collection. Each day, participants record what transport mode(s) they use to travel to and from work or study, the times they get up and go to bed, whether the accelerometer is removed for portions of the day, and if so, what activities the participant engages in during those times. No reliability or validity testing has been conducted with this tool. The information on waking hours and accelerometer removal derived from the log are checked and matched against accelerometer data.

#### • Body size measures

The interviewer measures body size at data collection 2. Height is assessed to the nearest 0.1 cm using a stadiometer (Mentone Educational Centre, Victoria, Australia) and weight to the nearest 0.1 kg using calibrated Seca 770 scales (Protec Solutions Ltd, Wellington, NZ). Body mass index (BMI) status for adults will be determined using the World Health Organization ethnic-specific thresholds [42,43] and the International Obesity Task Force criteria [44] will be applied to children. Waist circumference is measured as the minimum value between the iliac crest and the lateral costal margin (the mid-point between the hip and the lowest rib) to the nearest 0.1 cm using a Lufkin W606PM tape (Cooper Tools, Apex, NC, USA). Hip circumference is measured at the widest part of the buttocks [45]. Age-specific thresholds for high trunk mass will be applied to the sample [46-48].

#### • Neighborhood perceptions

Neighborhood perceptions are assessed using the Abbreviated-Neighborhood Walkability Scale (A-NEWS). The A-NEWS is a 54-item tool that measures adults' perceptions of dwelling density, land use mix, street connectivity, walking and cycling infrastructure, safety, and access to public and private facilities within their self-defined neighborhood. Responses are rated either on a four- or five-point Likert scales. Acceptable reliability and validity of the A-NEWS has been determined previously [49]. Neighborhood self-selection preferences are assessed on a five-point Likert scale using six items taken from the Strategies for Metropolitan Atlanta's Regional Transportation and Air Quality Study [50]. The neighborhood self-selection measures have also been used in the Neighborhood Quality of Life Study [51] and the Physical Activity in Localities and Community Environments [9]; these studies also contribute to the IPEN dataset.

#### • Self-reported PA

The IPAQ-LF is administered to capture adults' self-reported PA levels for the previous seven days (the period when the accelerometer was worn). The IPAQ-LF has shown to be a reliable and valid measure of PA engagement in 12 countries [40], and is used to assess PA engagement across four domains: occupational, transportation, household, and leisure. The outcome measures for overall and domain-specific PA will be frequency (days), duration (minutes), and intensity (light, moderate, and vigorous) of engagement. Self-reported PA levels will be compared with national PA recommendations, accelerometer data, and other countries participating in IPEN.

#### • Demographics

As part of the study, adult participants complete a demographic survey that examines: ethnicity, marital status, household income, academic qualifications, occupation, travel mode engagement, dwelling type, number of children living in the dwelling, time spent watching television, perceptions of body size, and the location of proximal and usually accessed food stores. Adult participants also complete the child's survey by proxy if an eligible child within the household participated in the study. Questions relating to the child include: ethnicity, screen time (e.g., television, computer, games consoles) access and rules, PA participation and motor skill ability, perceptions of body size, and access to and use of potential PA settings.

#### • Weather

Daily weather data (minimum and maximum temperature (°C), rainfall (mm)) are recorded at sites located in each of the four cities. The New Zealand Metrological Service collects and provides this information. Time-matched weather variables will be created to examine or control for the weather effects on PA engagement.

#### Phase 5: Personalized walkability measures

Personalized walkability index values will be calculated and constructed for adults and children based on the physical environment surrounding each participant's place of residence. The buffer distance will be developed along a one-kilometer street network from the participant's residence, excluding areas that cannot be accessed due to major barriers (e.g., freeways, water features). Similar GIS approaches as used to construct the walkability indices applied to the neighborhood selection process will be used to create the personalized walkability index classifications. Other potential inclusions in the index include public open space, public transport infrastructure, and topography variables within the buffer zone. Creation of these individualized measures has been conducted in previous research [52-54], and is a useful tool to enable the objectively measured built environment variables to be compared with individual-level health and self-report data.

#### Phase 6: Comparison of objective and self-report measures

International research suggests there is a mismatch between measures of perceived and objectively assessed PA facility availability [55] and behaviors [56], and these relationships require further investigation. Accordingly, it is important to examine the independent associations and levels of agreement between actual and perceived PA infrastructure at the neighborhood level within the New Zealand context, and the relationships between objective and perceived PA behaviors. Objective measures derived from GIS, the streetscape audit, accelerometers, and body size will be compared with self-report measures drawn from the photovoice and door-to-door data collection components of the study.

#### Phase 7: Multilevel modeling

Multilevel modeling is one of the more appropriate methods for understanding how multiple factors occurring at various hierarchical levels (such as individual, household, neighborhood, and city variables) operate to influence PA engagement and body size. The sampling frame and research design enables multilevel analyses of neighborhood environmental predictors for self-reported and objectively measured PA and body size for Māori and non-Māori adults and children. These analytic strategies appropriately accommodate and model the hierarchy and clusters within the research design, and allow for the adjustment of important potential confounders (such as rainfall). Further analyses will likely consider how the influence of parental variables impacts on child health behaviors at the household level.

#### Power calculations

Precise power calculations depend on focused and pre-determined statistical quantities; something that can be difficult for multi-aimed and broad studies such as this. For the purpose of this study, we intend recruiting 2,000 adults. However, a 10% reduction of our data is expected due to lack of compliance, reducing the data available for full analysis to 1,800 adults. Based on 12 background covariates explaining 25% of the variability of the dependent variable, and intraclass correlation coefficient cluster effects of 0.05, a realized sample of 1,800 adults, α = 0.05 and statistical power of 80%, the clustered multi-linear regression models will detect the smallest change in *r*^2 ^of ≤ 2.3% and clustered logistic regression models odds ratio of ≤ 1.27 if the prevalence rate of overweight/obesity is 60%. For the Māori and non-Māori comparisons, we expect lower Māori neighborhoods to have approximately 7% of the usual residents classified as Māori and higher Māori neighborhoods to have approximately 30% of the usual residents to identify as Māori [32]. Assuming a significant level of α = 0.05 and statistical power of 80%, then the detectable difference between any Māori and non-Māori proportion is within ± 10% for this sample size. A difference of ± 10% was considered epidemiologically worthwhile and important to detect.

## Discussion

Although characteristics of the built environment have been related to PA engagement [9,11,12,22] and obesity levels [6-8], the epidemiological understanding of the associations between urban form and health outcomes still remains largely unknown. Improved understanding of built environmental influences on health behaviors, through socio-ecological models, is needed to inform more effective and sustainable interventions [28]. The URBAN Study will contribute to the evidence base pertaining to PA engagement, body size, and the built environment for adults and children by overcoming some of the existing methodological limitations in this field.

### Applications of the URBAN Study

Four key research gaps in this area have been identified which the URBAN Study attempts to address. First, it is feasible that the limited environmental variability shown in urban locations previously investigated has underestimated the strength of associations between health outcomes and urban design [28]. The URBAN Study purposefully selects neighborhoods based on a diverse range of walkability and ethnicity characteristics, and contributes data to a multi-country study (IPEN). Second, although several studies have documented associations between the built environment and weight status [6,8] and PA engagement [57,58], confirmatory studies have yet to be conducted in diverse communities using robust measures to determine any walkability effect. Understanding these relationships in greater detail using standardized objective measurement procedures and protocols (GIS, accelerometers, body size) will provide more rigorous urban planning guidance to decision makers, thereby increasing the likelihood of improving population-level body size and PA outcomes. To our knowledge, this is the first New Zealand study to simultaneously use objective and self-report measurement tools to assess adult and child PA levels and body size status with the built environment. Third, limited evidence exists regarding how those individuals of different ethnicities, ages, genders, and/or family structures are influenced by the impact of neighborhood design with regard to health outcomes [28]. The URBAN Study has been designed to in part address this issue, with findings that can be stratified and analyzed according to these variables. Fourth, internationally there is very little evidence available identifying which built environment variables influence children's PA and body size, and how the built environment impacts on parental choices regarding children's PA opportunities. Accordingly, the URBAN Study will contribute directly to this evidence base by examining the interactions between children's PA behaviors, body size, parental perceptions, and built environment characteristics across diverse settings and child age ranges. It is anticipated that full results of the study will be available in 2011.

### Strengths and weaknesses of the URBAN Study

The obvious strengths of the URBAN Study are the: replication of international procedures and measures, neighborhood stratification and selection processes, use of objective and self-report measures, assessment of PA engagement over multiple domains, ability to control for seasonal effects, large sample size recruited, and incorporation of adults and children of diverse ethnicities into the sampling frame. Limitations of the study include its cross-sectional research design that means causality cannot be determined, and that neighborhoods are only drawn from urban settings; therefore findings cannot be applied to rural or small town environments within New Zealand. Neighborhood walkability and ethnicity classifications may also differ by region, and communities classified as being higher walkable or higher Māori population in one city may not reach the inclusion threshold for another city. However, this may also be considered a strength of the study as the design will allow any city-specific or dose-response effects to be captured, and assist with the understanding of the relative importance of other covariates and confounders. Lastly, neighborhoods are grouped according to geographic layout through contiguous mesh-blocks, rather than according to natural and social boundaries. This may create a mismatch between the GIS-assessed neighborhood and respondents' perceptions of their neighborhoods.

## Conclusion

Taken together, the URBAN Study will generate robust scientific evidence by using appropriate and standardized measures to provide a New Zealand-specific understanding of the associations between urban design and health outcomes, as well as contributing data to an international research project. Providing this information will impart urgently needed epidemiological information regarding the associations between the built environment and health outcomes. Accordingly, this study directly addresses the international priority issues of increasing PA engagement and decreasing obesity levels at the population-level.

## Abbreviations

A-NEWS: Abbreviated – Neighborhood Environment Walkability Scale; BMI: Body mass index; °C: Degrees Celsius; GIS: Geographical information systems; IPAQ-LF: International Physical Activity Questionnaire – Long Form; IPEN: International Physical Activity and Environment Network; mm: Millimeters; PA: Physical activity; SPACES: Systematic Pedestrian and Cycling Environment Scan; URBAN: Understanding the Relationships between Activity and Neighbourhoods.

## Competing interests

The authors declare that they have no competing interests.

## Authors' contributions

HMB developed the first draft of the manuscript. HMB, GMS, KW, PJS, SM, and RK contributed to the conception and the design of the study. All authors provided critical feedback during manuscript development. Each author has read and approved the final manuscript.

## Authors' information

HMB is a Post-doctoral Research Fellow at the Centre for Physical Activity and Nutrition Research, Auckland University of Technology, New Zealand. GMS is a Professor of Public Health and the Director of the Centre for Physical Activity and Nutrition Research, Auckland University of Technology, New Zealand. KW is an Associate Professor at the Centre for Social and Health Outcomes Research and Evaluation, Massey University, New Zealand. PJS is a Professor of Biostatistics at the School of Public Health and Psychosocial Studies at Auckland University of Technology, New Zealand and the School of Nursing and Midwifery, University of Queensland, Australia. SM is a GIS Analyst at the Centre for Social and Health Outcomes Research and Evaluation, Massey University, New Zealand. RAK is a Professor of Geography at the School of Geography, Geology, and Environmental Sciences, University of Auckland, New Zealand. EAH is the Head of Research at the School of Sport and Recreation at Auckland University of Technology, New Zealand. MO is a Post-doctoral Research Fellow at the Centre for Physical Activity and Nutrition Research, Auckland University of Technology, New Zealand. HK is a researcher at Whariki Research Group, Massey University, New Zealand. VGJ is a researcher at Whariki Research Group, Massey University, New Zealand. CE is a PhD student at the School of Geography, Geology, and Environmental Sciences, University of Auckland, New Zealand. LM is a PhD student at the Centre for Physical Activity and Nutrition Research, Auckland University of Technology, New Zealand. JM is the Research Manager at the Centre for Physical Activity and Nutrition Research, Auckland University of Technology, New Zealand

## Pre-publication history

The pre-publication history for this paper can be accessed here:


